# Role of Heart Rate Variability in the Association between Myocardial Infarction Severity and Post-Myocardial Infarction Distress

**DOI:** 10.3390/life13122266

**Published:** 2023-11-27

**Authors:** Reham Dyab, Claudia Zuccarella-Hackl, Mary Princip, Sinthujan Sivakumar, Rebecca E. Meister-Langraf, Hansjörg Znoj, Jean-Paul Schmid, Jürgen Barth, Ulrich Schnyder, Roland von Känel, Yori Gidron

**Affiliations:** 1 The Cheryl Spencer Department of Nursing, Faculty of Social Welfare and Health Science, The University of Haifa, Haifa 3498838, Israel; 2 Lady Davis Carmel Medical Center, Haifa 3436212, Israel; 3 Department of Consultation-Liaison Psychiatry and Psychosomatic Medicine, University Hospital Zürich, 8091 Zurich, Switzerland; claudia.hackl-zuccarella@usz.ch (C.Z.-H.); mary.princip@usz.ch (M.P.); sinthujan.sivakumar@usz.ch (S.S.); rebecca.langraf-meister@usz.ch (R.E.M.-L.); roland.vonkaenel@usz.ch (R.v.K.); 4 Faculty of Medicine, University of Zürich, 8006 Zurich, Switzerland; ulrich.schnyder@access.uzh.ch; 5 Clienia Schlössli AG, 8618 Oetwil am See, Switzerland; 6 Department of Clinical Psychology and Psychotherapy, University of Bern, 3012 Bern, Switzerland; hansjoerg.znoj@psy.unibe.ch; 7 Department of Internal Medicine and Cardiology, Clinic Gais AG, 9056 Gais, Switzerland; jean-paul.schmid@kliniken-valens.ch; 8 Institute for Complementary and Integrative Medicine, University Hospital Zürich, University of Zürich, 8006 Zurich, Switzerland; juergen.barth@usz.ch

**Keywords:** myocardial infarction, stress disorders, post-traumatic symptoms, depressive signs and symptoms, heart rate determination, moderation

## Abstract

Objective: Myocardial infarction (MI) results in mental health consequences, including depression and post-traumatic stress disorder (PTSD). The risk and protective factors of such mental consequences are not fully understood. This study examined the relation between MI severity and future mental health consequences and the moderating role of vagal nerve activity. Methods: In a reanalysis of data from the Myocardial Infarction-Stress Prevention Intervention (MI-SPRINT) study, 154 post-MI patients participated. MI severity was measured by the Killip Scale and by troponin levels. Depression and PTSD symptoms were assessed with valid questionnaires, both at 3 and 12 months. Vagal nerve activity was indexed by the heart rate variability (HRV) parameter of the root-mean square of successive R-R differences (RMSSD). Following multivariate analyses, the association between MI severity and distress was examined in patients with low and high HRV (RMSSD = 30 ms). Results: In the full sample, the Killip index predicted post-MI distress only at 3 months, while troponin predicted distress at 3- and 12-months post-MI. However, HRV moderated the effects of the Killip classification; Killip significantly predicted symptoms of depression and PTSD at 3- and 12-months post-MI, but only in patients with low HRV. Such moderation was absent for troponin. Conclusion: MI severity (Killip classification) predicted post-MI depression and PTSD symptoms, but only in patients with low HRV, suggesting that the vagal nerve is a partial protective (moderating) factor in the relation between Killip score and post-MI distress.

## 1. Introduction

Myocardial infarction (MI) is a life-threatening event that not only causes physiological and cardiac damage, but also has psychological consequences such as depression and post-traumatic stress disorder (PTSD) [1,2,3,4]. Depressive symptoms include negative affect such as deep sadness, negative cognitions about oneself or the world, reduced activity levels, and general anhedonia. PTSD symptoms include flashbacks and sensory reliving of the event, attempts to avoid thoughts and places related to the event, physiological arousal such as poor sleep and concentration difficulties, and, finally, negative affect and thoughts about oneself and the world [5]. Depression after MI is not just related to poor quality of life in these patients, but most importantly, it predicts a higher risk of mortality [2,4,6]. Similarly, PTSD is related to worse quality of life and a higher risk of recurrent cardiac events [7].

Systematic reviews and meta-analyses have revealed that there is wide variability in the prevalence rates of post-MI depression, ranging from 13.5% to 41.6%. This wide variability may be related to differences in the tools used to assess depression [8,9]. Similarly, the mean prevalence of clinically significant post-MI PTSD symptoms was reported in a meta-analysis to be 12% and depended upon the type of assessment (self-reported questionnaires versus structured diagnostic clinical interviews) [10].

Risk factors for post-MI depression include young age, female gender, hypercholesterolemia, the use of certain medications (e.g., calcium channel blockers), and low left ventricular ejection fraction (LVEF) [11]. Risk factors for post-MI PTSD include young age, female gender, prior psychiatric history, prior trauma, subjective feelings of life threat during MI, acute stress symptoms during MI, and low social support [12].

In MI, there are several indexes of disease severity that predict cardiovascular prognosis. These include troponin T, Killip class, and LVEF. Troponin represents the necrosis of cardiac cells [13] and is an independent predictor of mortality [14]. The Killip class indicates the severity of heart failure in MI patients with a higher-class mortality prediction for ST-elevation MI (STEMI) and non-STEMI [15]. LVEF is a measure of left ventricular function expressed in percent, and an LVEF < 25% is an independent predictor of post-MI mortality [16].

Few studies have examined if MI severity is a risk factor of depression and PTSD symptoms. One study showed that reduced LVEF predicted post-MI depression [11]. But Killip class and troponin levels represented more direct measures of MI severity. 

One common neurophysiological factor, which could affect disease severity on the one hand and post-MI distress on the other, is the vagal nerve. The vagal nerve is the main branch of the parasympathetic nervous system, involved in the functioning of many body organs and systems, including the heart. Activity of the vagal nerve is indexed by heart rate variability (HRV). While HRV is not equal to actual vagal nerve activity, the two highly correlate (r = 0.88), and vagomimetic medication increases HRV [17]. Time-domain measurements are used to calculate the root-mean square of successive differences (RMSSD), which is less influenced by breathing, and RMSSD is considered a valid index of vagal nerve activity. Finally, this index was found to differentiate individuals with and without primary risk factors [18]. For these reasons, we focused on RMSSD in the present study.

High HRV predicts a 4-fold increased post-MI survival according to a meta-analysis of 21 studies [19]. In addition, low HRV has been quite consistently related to depression [20,21]. Similarly, a meta-analysis of 43 studies found lower HRV in patients with PTSD [22]. Several mechanisms exist to link vagal activity to better post-MI prognosis and a lower risk of distress, including lower sympathetic activity and reduced inflammation [23,24]. Indeed, the vagal nerve is a neuro-immuno-modulator since it inhibits inflammation with hormonal cortisol and neural–splenic routes [25]. HRV was found to be a moderator of biological systems and disease course in several occasions. For instance, HRV determined the velocity of physiological recovery from stress [26]. In cancer patients, HRV moderated the effects of the disease stage on tumor marker levels [27]. From this literature, the question arises whether HRV can moderate the effects of MI severity on post-MI distress. Therefore, the purpose of this study was to examine the moderating role of vagal activity in the relationship between MI severity and both post-MI depression and MI-induced PTSD symptoms. We hypothesized that only in patients with low HRV would MI severity predict mental distress, while in patients with high HRV, MI severity would be less strongly related to mental distress in the aftermath of MI.

## 2. Methods

### Study Design and Participants

This was a secondary analysis of the Myocardial Infarction-Stress Prevention Intervention (MI-SPRINT) randomized controlled trial, which investigated the effectiveness of one single session of early psychological counselling on the prevention of MI-induced PTSD symptoms [28]. The study was carried out in accordance with the Declaration of Helsinki and the guidelines on Good Clinical Practice and was approved by the Ethics Committee of the State of Bern (KEK No. 170/12). The study protocol was registered under ClinicalTrials.gov (NCT01781247) and independently monitored by the Clinical Trials Unit, the Faculty of Medicine, University of Bern. Informed consent was obtained from all subjects involved in the study. Participants did not receive financial compensation. 

The MI-SPRINT trial enrolled 190 Caucasian patients with verified STEMI or non-STEMI, referred for acute coronary intervention to the Cardiology Department, Bern University Hospital, Switzerland, between 2/2013 and 9/2015. Participants were randomly assigned to either the trauma-focused intervention group (n = 97) or the stress-focused active control group (n = 93). Of the 190 participants enrolled at admission, 154 participated in the 3-month follow-up and 106 participated in the 12-month follow-up investigation. Recruitment details, reasons for dropouts, and the content of the intervention have been described elsewhere [29]. 

Consecutive patients were 18 years or older with stable circulatory conditions and a high level of distress during MI, assessed by the numeric rating scale (range 0–10) for ‘pain intensity’ (during MI), ‘fear of dying’ (until admission to the coronary care unit), and/or ‘worrying and feeling helpless’ (when being informed about having MI). Patients were excluded if they received emergency coronary artery bypass grafting, if they had comorbid diseases likely to cause death within 1 year, were not fully orientated, or had cognitive impairment. Further exclusion criteria included current severe depression in the patient’s history as well as, based on the cardiologist’s clinical judgment, an insufficient knowledge of German to follow the study instructions, and participation in another randomized controlled trial. 

## 3. Measures

### 3.1. Heart Rate Variability—HRV

Using a TNO Biomedical Instrument Finometer device, Amsterdam, The Netherlands, The Netherlands, continuous recordings on the finger were obtained from each patient during 5-min intervals while resting in a stable supine position [30]. The Finometer recorded the variability and short-term variations in blood pressure as well as the beat-to-beat finger pulse contour. On a hard drive, all cardiovascular variables were digitally saved in result files. The participant’s sex, age, and body mass index (BMI) were integrated with the HRV values using Beatscope 1.1a software [31]. Inter-beat intervals (IBI in milliseconds) were exported using Beatscope 1.1a to a single text file. Within 12–24 h of admission to the Critical Care Unit (CCU), patients were informed about the planned study. If the eligibility criteria were fulfilled and after having provided informed consent, HRV was measured. Frequency and time-domain measures of HRV were measured for 5 min with an electrocardiogram recording during stable supine resting using customized Beatscope 1.1a software program (Finometer TPD Biomedical Instruments, Amsterdam, The Netherlands). Afterward, the counseling session for 45 min took place (as this was a reanalysis of an intervention trial). Fasting blood samples were collected the next morning at 6 a.m. The export of the IBIs to an Excel sheet was done in a subsequent stage, where clear artifacts (IBI > 1800 ms; IBI < 300 ms) were found and manually excluded. The Biosignal Analysis and Medical Imaging Group (BSAMIG; http://kubios.uef.fi/Kubios accessed on 31 August 2022) HRV analysis package 2.2 was used to evaluate and perform time-domain measurements of the cardiac period power spectrum. By using cubic spline interpolation to replace the lost IBIs, we were able to correct artifacts with Kubios HRV. We spectrally measured the HRV power (in milliseconds squared per hertz). The heart rate (HR) time series divided the HR variation into spectral components and calculated their power using frequency domain methods [31]. 

Time-domain measurements were used to calculate the root-mean square of successive differences (RMSSD). RMSSD is considered a valid index of vagal nerve activity. To achieve a normal distribution, RMSSD values were logarithmically transformed (base 10) for the analysis [32]. 

### 3.2. Severity of Myocardial Infarction

The Severity of Myocardial Infarction was indexed with Killip class and troponin levels. Killip Classification: the Killip classification, a well-recognized clinical assessment tool, was used to assess heart failure severity in patients with acute MI. According to the Killip and Kimball criteria, patients are classified into four classes based on findings of physical examination: Class I: no evidence of heart failure; Class II: findings consistent with mild to moderate heart failure (e.g., S3 gallop, lung rales less than one-half way up the posterior lung fields, or jugular venous distension); Class III: overt pulmonary edema; and Class IV: cardiogenic shock [33]. Studies have shown that the Killip classification is a significant predictor of short- and long-term mortality in patients with STEMI and non-STEMI, as well as in patients with unstable angina [34,35]. 

Troponin represents levels of myocyte cell death and is an independent predictor of post-MI prognosis [36]. 

### 3.3. Psychological Assessment

Post-traumatic stress symptoms: the validated self-rating post-traumatic diagnostic scale (PDS), German version, was used to assess PTSD symptom severity at the 3- and 12-month follow-ups [37]. The PDS measures the three core symptoms of PTSD: (1) re-experiencing, (2) avoidance/numbing, and (3) hyperarousal, according to the Diagnostic and Statistical Manual of Mental Disorders, Fourth Edition (DSM-IV). It consists of 17 items, which are scored on a 4-point Likert scale ranging from 0 (not at all or only once) to 3 (five or more times a week/almost always). The total severity score ranged between 1–10, 11–20, 21–35, and 36–51, indicating mild, moderate, moderate-to-severe, and severe PTSD symptoms, respectively [38]. Cronbach’s α was 0.81 for the total PTSD score in the current study. Depressive symptom severity: depressive symptom severity was measured using the validated German version [39] of the Beck Depression Inventory-Second Edition (BDI-II; [40]), which consists of 21 items. The BDI was developed for the assessment of depressive symptoms that correspond to the DSM-IV criteria for major depressive disorders and measures a somatic and a cognitive–affective dimension of depression [40]. The BDI-II assesses the frequency and/or severity of symptoms related to sadness, feelings of guilt, perceptions of self-worth, suicidal ideation, and changes in appetite and body weight, among other characteristics. Items are scored on a 4-point Likert scale, ranging from 0 (symptoms not present) to 3 (symptoms very present). The BDI-II total score ranges from 0 to 63, with higher scores reflecting higher depressive symptom severity. Cronbach’s α of the BDI-II total score was between 0.84 and 0.91 for non-psychiatric populations [39], and was 0.66 for depression in our sample. 

### 3.4. Statistical Analysis

The Killip class was divided into two categories (1 vs. 2–4) due to the small number of patients in some of the classes. The main analyses were performed with Pearson correlations to examine the associations between MI severity and post-MI distress levels for troponin levels only. To test the moderating role of HRV, we examined the interactions between MI severity and HRV using hierarchical multiple regressions. In these analyses, MI severity (troponin) was the predictor, HRV was the moderator, and PTSD and depression symptom scores were the outcomes. After observing interaction effects, we performed a multiple hierarchical linear regression for mental distress, where in block 1, we entered age, gender, group of treatment, and MI type, and in block 2, we separately entered troponin for patients with low and high HRV. The adjusted variables were chosen according to their role in mental distress from the literature and if they were significantly related to each outcome. Interactions were also followed by partial correlation analyses between MI severity and mental distress, separately performed in patients with high and low HRV. Such an analysis was justified by past studies that showed that associations between different biomarkers and clinical outcomes were different in patients with high versus low HRV [18,26,41]. 

For Killip classification, we tested the main effects of Killip and the Killip * HRV interaction using an analysis of co-variance (ANCOVA), where we categorized both Killip (1 vs. >1) and HRV (RMSSD < 29 vs. RMSSD > 29) and statistically controlled for confounders. This cutoff of RMSSD was near our sample median and was close to the cutoff proposed in a large-scale study [18].

## 4. Results

The sample included 157 men (82.6%) and 33 women (17.4%); 51.1% were in the experimental group and 49.9% in the control group. The age range was 18–88 years. Concerning the MI type, 71.3% had STEMI and 28.7% had non-STEMI. Concerning Killip classification, 82.5% had Class I, 10.6% had Class II, 0.5% had Class III, and 6.3% had Class IV. Concerning LVEF, the mean (SD) was 47.6% (11.9) with a range of 20–75%. Table 1 shows the descriptive statistics of the continuous variables. The mean age of the sample was 60 years, and the mean RMSSD was below 42, which reflected the mean of healthy people [42]. 

### Relationship between Disease Severity and Mental Health

Medications such as statins, beta blockers, and anti-coagulants, as well as age, gender, previous MI, and type of MI, were not related to post-MI distress (all *p* > 0.05). We nevertheless controlled for the effects of age, gender, diabetes, previous MI, and type of MI in the multivariate analysis because of the known roles in the literature.

In the full sample, the Killip class significantly and positively predicted depressive symptoms at 3 months (F (1,135) = 9.83, *p* = 0.002) and PTSD symptoms at 3 months (F (1,136) = 6.37, *p* < 0.013). In contrast, the Killip class did not significantly predict depression (F (1,93) = 0.49, *p* = 0.49) or PTSD symptoms (F (1,93) = 0.83, *p* < 0.36) at 12 months post-MI. Using a linear multiple regression, we then examined the unique contribution of troponin levels in predicting post-MI distress, controlling for confounders. Controlling for age, gender, past MI, having STEM or not, and group, troponin accounted for an additional and significant 5.7% of the variance in post-MI PTSD symptoms at 3 months (F1,136) = 8.76, *p* = 0.004) and accounted for an additional and significant 6.0% of the variance in post-MI PTSD symptoms at 12 months (F1,93) = 6.10, *p* = 0.015). Controlling the above confounders, troponin accounted for an additional and significant 5.3% of the variance in post-MI depression symptoms at 3 months (F1,135) = 8.12, *p* = 0.005) and accounted for an additional and significant 5.3% of the variance in post-MI depression symptoms at 12 months (F (1,93) = 5.33, *p* = 0.02). 

RMSSD was not significantly correlated with the Killip score (r = −0.05, *p* = 0.56) or with troponin levels (r = 0.15, *p* = 0.08).

Interestingly, groups II and IV had the worst cardiologic and psychological profiles. In Killip I (n = 111), the mean HRV was 37.75, (sd = 37.40). In Killip II (n = 13), the mean HRV was 33.27, (sd = 21.18), and in Killip IV (n = 8), the mean HRV was 31.52 (sd = 13.41). However, the number of participants in some Killip subgroups was too small for group comparisons with meaningful statistical power. The Killip category was unrelated to HRV (F (2,129) = 0.19, *p* > 0.05).

**Figure 1:**
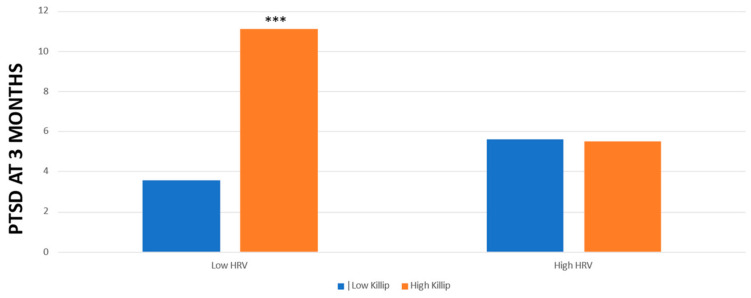
Levels of PTSD at 3 months post-MI in patients with low/high Killip and low/high HRV. *** *p* < 0.001.

We then examined the interaction between HRV and Killip in relation to each outcome with an ANCOVA. Concerning PTSD symptoms at 3 months, the Killip * HRV interaction was significant (F (1,97) = 6.55, *p* = 0.012) after controlling for group treatment, age, gender, MI type, and previous MI. Figure 1 shows these results. 

**Figure 2:**
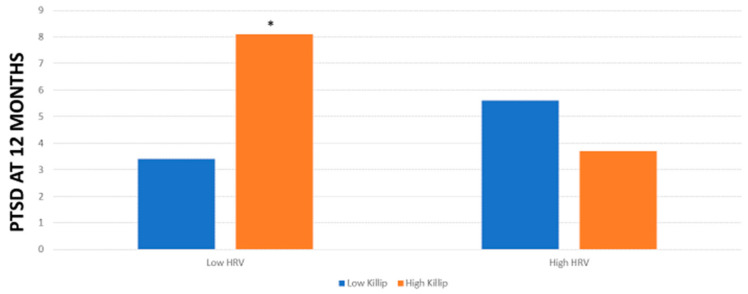
Levels of PTSD at 12 months post-MI in patients with low/high Killip and low/high HRV. * *p* < 0.05.

Concerning PTSD symptoms at 12 months, the Killip * HRV interaction was also significant (F (1,63) = 5.42, *p* < 0.02) after controlling for all confounders. Figure 2 shows these results.

**Figure 3:**
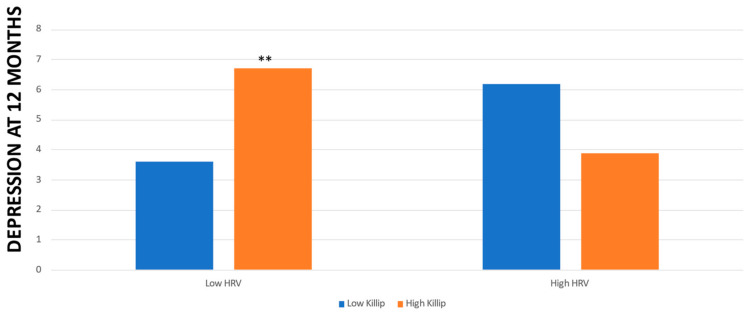
Levels of depression at 12 months post-MI in patients with low/high Killip and low/high HRV. ** *p* < 0.01.

Finally, concerning depressive symptoms at 12 months, the Killip * HRV interaction was significant (F (1,63) = 6.35, *p* = 0.01) after controlling for all confounders (Figure 3 shows these results).

Following these interactions, in patients with low HRV, we found that Killip class significantly predicted PTSD symptoms at 3 months (F (1,49) = 13.56, *p* < 0.001). In patients with high HRV, Killip class did not predict PTSD at 3 months (F (1,453) = 0, *p* = 0.96). Concerning PTSD symptoms at 12 months in patients with low HRV, we found that Killip class significantly predicted PTSD symptoms (F (1,25) = 5.96, *p* = 0.02). However, in patients with high HRV, Killip class did not predict PTSD symptoms (F (1,33) = 0.41, *p* = 0.53). 

Concerning depressive symptoms at 3 months, there was no need to search for the source of the interaction since HRV * Killip was not significant. Concerning depression symptoms at 12 months, in patients with low HRV, we found that Killip class significantly predicted depression symptoms (F (1,25) = 8.09, *p* = 0.009). In contrast, in patients with high HRV, Killip class did not predict depression symptoms (F (1,33) = 1.29, *p* = 0.26).

We then examined the interaction between HRV and troponin in relation to each outcome with a hierarchical multiple regression. Concerning PTSD symptoms at 3 months, the troponin * HRV interaction explained an additional and nearly significant 3.2% of the variance after controlling for all confounders and the main effects of troponin and HRV (F (1,97) =3.55, *p* = 0.06). Though not significant, we examined the source of this interaction. The correlation between troponin and PTSD at 3 months in patients with low RMSSD was r = 0.06 (*p* = 0.67), and in patients with high RMSSD, r = 0.28 (*p* = 0.06), controlling for confounders. Concerning PTSD symptoms at 12 months, the troponin * HRV interaction explained an additional and non-significant 1.2% of the variance, after controlling for all confounders (F (1,63) = 0.84, *p* = 0.36). Concerning depression symptoms at 3 months, the troponin * HRV interaction explained 0% of the variance, after controlling for all confounders (F (1,97) = 0.03, *p* = 0.87). Finally, concerning depression symptoms at 12 months, the troponin * HRV interaction explained 0% of the variance, after controlling for all confounders (F (1,63) = 0.03, *p* = 0.86).

## 5. Discussion

In this study, we aimed to first test the relationship between the severity of MI and mental health outcomes 3- and 12-months post-MI. Second, we examined the moderating role of HRV in the relationship between MI severity and post-MI depression and PTSD symptoms. In the full sample, we found that the Killip class significantly and positively predicted depression and PTSD symptoms at 3 months. In contrast, the Killip class did not predict depression or PTSD symptoms at 12-months post-MI. Troponin levels predicted both post-MI depression and PTSD symptoms at 3 and 12 months, independent of confounders. 

Importantly, HRV was identified as a moderator in these effects, especially for Killip class. Specifically, we found that the Killip class significantly and positively predicted both depression and PTSD symptoms at 3 and 12 months, but only in patients with low HRV. Killip class did not predict depression or PTSD symptoms at 3 or at 12 months in patients with high HRV. Controlling for effects of treatment group, age, sex, previous MI, and STEMI did not change these associations, attesting to the robustness of our findings. In contrast to Killip, HRV did not significantly moderate the relations between troponin and post-MI PTSD and depression symptoms, and only a trend was found for this in relation to PTSD at 3 months.

A previous meta-analysis revealed that post-MI depression was associated not only with reduced quality of life but also with worse prognosis [43]. Similarly, post-MI PTSD was related to a two-fold increased mortality or recurrence risk [10]. Hence, it is crucial to identify the predictors of post-MI distress and particularly of post-MI depression and PTSD symptoms. In the present study, we found the Killip class and troponin levels to significantly and positively predict post-MI depression symptoms at 3 months and PTSD symptoms at 3 months. Troponin also predicted these outcomes at 12 months. Similar results were found in other studies showing associations between disease severity (e.g., Killip class) and depressive symptoms [11]. Another study found that low LVEF was correlated with an incidence of post-MI depression [44]. In another study, lower LVEF was an independent predictor of anxious mood and emotional problems 3 months after discharge from hospital [45]. However, the present study extended these findings and found that patients’ initial vagal nerve activity (RMSSD) moderated the association between disease severity (Killip class) and post-MI distress. Only in patients with low HRV did MI severity (Killip class) predict depression and PTSD symptoms. Such a relationship was absent when RMSSD was high. In order to avoid a type-1 error, we focused our analyses on the HRV index of RMSSD since it is the direct measure of vagal activity and since it is less sensitive to breathing cycle changes. Future studies should try to replicate our findings using additional indexes of HRV.

Our results echoed those of a previous study in which high HRV moderated the effects of stress on hormonal (cortisol), cardiovascular (DBP), and inflammatory (TNF) stress responses, and high HRV predicted faster biological recovery from stress [26]. Moreover, HRV measures are strongly associated with morbidity and mortality in various diseases. High HRV was found in a meta-analysis of 21 studies to predict on average a 4-times higher chance of survival in post-MI patients [46], and high HRV predicted a higher survival rate in cancer as well [46]. Furthermore, depressive states are associated with low HRV [47], and PTSD symptoms are also related to low HRV [48]. As HRV is strongly and positively correlated with actual vagal nerve activity (r = 0.88) [17], these results call for further research to investigate the mechanisms of vagal neuromodulation mitigating adverse effects of MI severity on mental distress. One possibility is that a worse MI may reduce physical activity, which is related to lower HRV, which then could result in more negative mental states. Another mechanism could be that a more severe MI may induce more inflammation, which results in more severe mental distress [49]. These could be biologically moderated by the anti-inflammatory actions of the vagus [25] if the vagus activity were improved, as mentioned above. The veracity of these proposed mechanisms requires further investigation.

One may wonder why HRV moderated only the predictive effects of Killip class and not of troponin, in relation to post-MI distress. Troponin more directly reflects the actual amount of myocyte cellular death, which may be difficult to override by stronger vagal activity. In contrast, the Killip class reflects disability to a greater extent than troponin, and thus, Killip class may have more of a behavioral impact. Given the bridging role of the vagus between the brain and the body, HRV, the vagal index, may moderate such a relationship between Killip class and distress, associated with disability.

This study had several limitations that should be mentioned. First, the study was conducted in the context of an RCT. However, effects of the therapeutic group were statistically controlled for. Second, our main observation was based on a correlational design. Future randomized clinical trials should examine whether increasing vagal nerve activity prevents post-MI depression and PTSD symptoms in patients with a high Killip class to show whether our observation was causal and of clinical significance. Third, the measurement of HRV was obtained from a Finometer device using pulse waves, which could have included ectopic beats specific to post-MI patients. However, there was a high correlation between finger measures and ECG measures of HRV [48] and for SDNN also in cardiac patients [50]. Fourth, in an additional analysis, with HF HRV, the pattern of the results was different (the Killip class determined with HF interactions were mostly not significant). Since we did not show this pattern of results with other HRV parameters “e.g”, HF HRV or respiratory sinus arrythmia (RSA), future studies should replicate these findings. Furthermore, the correlation between RMSSD and HF was not strong (r = 0.34, *p* < 0.001) as would be expected. This was surprising and calls for further investigation and replication. Finally, we used a PTSD scale that was based on DSM-IV criteria, and future studies should replicate these findings using assessment tools based on the DSM-5. 

## 6. Conclusions

Despite these limitations, the protective role of HRV (RMSSD) was consistent in relation to two mental health outcomes over two different follow-up times, independent of confounders, which attest the robustness of these findings. These new results revealed a potential protective role of the vagal nerve in the adverse mental effects of MI severity, a role in line with the general protective role of this 10th cranial nerve in health and disease [50]. From a clinical standpoint, providing psychological treatment for patients with high Killip and low HRV as a risk group may be helpful to prevent post-MI distress.

## Figures and Tables

**Table 1:** Participant characteristics.

Variable	Mean	SD
Age (years)	59.9	11.2
LVEF, %	47.6	11.9
Troponin	3.94	4.84
PTSD symptoms at 3 Months	5.36	6.26
PTSD symptoms at 12 Months	4.94	6.00
Depressive symptoms at 3 Months	5.43	5.09
Depressive symptoms at 12 Months	5.30	4.58
RMSSD (msec)	36.9	35.1
Variable	Percentage (%)	
Gender		
Men	82.6	
Women	17.4	
MI Type		
STEMI	71.3	
Non-STEMI	28.7	
Killip class		
Killip I	82.5	
Killip II	10.6	
Killip III	0.5	
Killip IV	6.3	
Comorbidities		
Past depression	28.3	
Diabetes	14.5	
Hypertension	51.3	

Note: SD = standard deviation; LVEF = left ventricular ejection fraction, KILLIP = Killip classification, PTSD = post-traumatic stress disorder; RMSSD = route mean square of successive differences.

## Data Availability

Data are available upon request.
